# Gum-Saline in Wound-Shock and Similar States

**Published:** 1919-05-17

**Authors:** H. Robinson


### THE EDITOR'S LETTER-BOX.

**[Correspondence on all subjects is invited, but we cannot in any way be responsible for the opinions expressed by our correspondents, who must give their name and address as a guarantee of good faith, but not necessarily for publication. Correspondents are reminded that brevity of style and conciseness of statement greatly facilitate early insertion.]**

### Gum-Saline in Wound-Shock and Similar States.

*To the Editor of* The Hospital.

Sir,—Between September 1917 and February 1919 I worked in two base hospitals and two C.C.S., thus seeing both the immediate and the postponed surgery. Owing to the adoption of the team system I also met and worked with the surgical specialists of at least eight other base hospitals and C.C.S., to say nothing of surgeons other than specialists. I never met one who thought gum as good as blood for transfusion in combating the effects of shock or of hæmorrhage, though most of them had tried both (and given up gum). This is not to say that gum is useless: if blood cannot be got, gum may be a useful substitute. But to suppose that gum is comparable in any way with blood is an opinion which is certainly not held by those who were actively employed in France.—I am, Yours faithfully,

May 10, 1919. H. Robinson, M.D.

